# Reverse engineering highlights potential principles of large gene regulatory network design and learning

**DOI:** 10.1038/s41540-017-0019-y

**Published:** 2017-06-22

**Authors:** Clément Carré, André Mas, Gabriel Krouk

**Affiliations:** 1 Institut Montpelliérain Alexander Grothendieck, Université de Montpellier, Montpellier, France; 2 Laboratoire de Biochimie et Physiologie Moléculaire des Plantes, Institut de Biologie Intégrative des Plantes ‘Claude Grignon’, UMR5004 CNRS, INRA, SupAgro, UM, Place Pierre Viala, Montpellier, 34060 France

## Abstract

Inferring transcriptional gene regulatory networks from transcriptomic datasets is a key challenge of systems biology, with potential impacts ranging from medicine to agronomy. There are several techniques used presently to experimentally assay transcription factors to target relationships, defining important information about real gene regulatory networks connections. These techniques include classical ChIP-seq, yeast one-hybrid, or more recently, DAP-seq or target technologies. These techniques are usually used to validate algorithm predictions. Here, we developed a reverse engineering approach based on mathematical and computer simulation to evaluate the impact that this prior knowledge on gene regulatory networks may have on training machine learning algorithms. First, we developed a gene regulatory networks-simulating engine called FRANK (Fast Randomizing Algorithm for Network Knowledge) that is able to simulate large gene regulatory networks (containing 10^4^ genes) with characteristics of gene regulatory networks observed in vivo. FRANK also generates stable or oscillatory gene expression directly produced by the simulated gene regulatory networks. The development of FRANK leads to important general conclusions concerning the design of large and stable gene regulatory networks harboring scale free properties (built ex nihilo). In combination with supervised (accepting prior knowledge) support vector machine algorithm we (i) address biologically oriented questions concerning our capacity to accurately reconstruct gene regulatory networks and in particular we demonstrate that prior-knowledge structure is crucial for accurate learning, and (ii) draw conclusions to inform experimental design to performed learning able to solve gene regulatory networks in the future. By demonstrating that our predictions concerning the influence of the prior-knowledge structure on support vector machine learning capacity holds true on real data (*Escherichia coli* K14 network reconstruction using network and transcriptomic data), we show that the formalism used to build FRANK can to some extent be a reasonable model for gene regulatory networks in real cells.

## Gene Regulatory Networks: design and learning principles

This work by Carré et al addresses central questions in biology, which are: how very large gene regulatory networks (GRNs) are organized, generate stable gene expression, and can be learnt using machine learning algorithms? In this work authors developed an algorithm able to simulate large GRNs. From these networks they simulate stable or oscillating gene expression and highlights some mathematical rules controlling such a collective (several thousands of genes) behavior. They discuss consequent hypothesis concerning the organization of GRNs in real cells. Using this simulation tool, authors also demonstrate that it’s likely possible to computationally learn GRNs from transcriptomic data and prior knowledge on the network (actual known connections issued from Yeast One Hybrid or ChIP Seq for instance). They particularly highlight the crucial importance of the prior knowledge structure in their capacity to learn large GRNs.

## Introduction

Gene regulation plays a key role in the control of fundamental processes in living organisms, ranging from development, to nutrition and metabolic coordination. Genes are regulated at several levels of integration but one key step is the control of gene transcription. Determining the fundamental structure of transcriptional Gene Regulatory Networks (GRNs, considered here as the relationships of transcription factors (TFs) and their targets) is a major challenge of systems biology.^1–4^ Understanding GRNs has tremendous implications ranging from medicine to agriculture. Indeed, being able to learn GRNs may enable manipulating the cell as a system and potentially control and coordinate many physiological events that are related to GRN activity (diseases, biotechnological applications, crop production, and more). The quest of systems biology is thus to determine GRN structure using machine-learning algorithms applied on transcriptomic datasets [considered as the most exhaustive level measurement of the system to date (commonly assayed by microarrays or next generation sequencing)].

Furthermore, recent high throughput experimental approaches are now drafting the GRNs backbones for many different species,^5^ ranging from prokaryotes, yeast, plants to humans. We can distinguish two complementary types of approaches. The first type is TF-centered, such as Chromatin Immuno-precipitation followed by high-throughput sequencing [ChIP-seq, DAP-seq^6–12^ or TARGET procedures [Transient Assay Reporting Genome-wide Effect of TF].^13–16^ In these cases, one aims at investigating the binding activity of a particular TF across the genome or its capacity to activate its targets upon entrance in the nucleus. The second type of approach is target-centered, such as enhanced yeast-one hybrid (eY1H) approaches that decipher GRNs controlling a particular set of genes.^17–22^ These approaches, TF-centered and target-centered, can be understood as the closest proxy to experimentally determining actual GRN in living organisms.^23^

Interestingly, these experimental data on GNR connections are often used to validate algorithms predictions, but it can also be used as potential knowledge to train machine-learning procedures.^24^ Hence, the purpose of the current work is to understand: (i) how valuable is this GRN prior-experimental-knowledge, (ii) which characteristics of this prior-knowledge are potentially better in training or supervising machine-learning procedure to learn large GRNs from transcriptomic data? In other words can we, in the near future, possibly train algorithms to decipher real regulatory connections by combining ChIP-seq, DAP-seq, eY1H, or TARGET results with transcriptomic datasets?

Since no GRN is known with sufficient precision to be used as gold standard, we undertook a reverse engineering path. Indeed, training machine learning algorithms on real biological networks poses fundamental problems because these networks are not perfectly defined. This kind of approach is now routinely used, in particular during the DREAM challenges [http://dreamchallenges.org/; Dialog on Reverse Engineering Assessment and Methods].^3, 25–27^ This work demonstrated that learning GRNs even from in silico simulated transcriptional data is not trivial but can still provide significant results. During the several DREAM challenges that focused on GRNs inference, the machine learning procedures are trained on simulated gene expression, on mutant versions of the networks, as well as on perturbed networks, where expression of several genes is modified to simulate external influencing factors. Here, we use an approach which is quite different, since as mentioned above, we focus on using experimentally probed TF→ target as prior-knowledge. Our rationale is very close to what has been proposed before by Cerulo and colleagues,^24^ but quite different regarding the size of the simulated networks as well as the biological questions that we ask and answer.

Indeed, we decided to develop our own GRN-simulating algorithm called FRANK for Fast Randomizing Algorithm for Network Knowledge, which is able to (i) simulate very large networks (potentially containing as many genes as real eukaryotic genomes ~10^4^ genes including ~10^3^ TFs); (ii) simulate gene expression over several thousands of simulated time points or system levels (see below), (iii) in a relatively short computation time (several minutes). The decision to work on very large networks comes with trade-off concerning mathematical formalism fully discussed thereafter. Indeed, it is worth noting that several network simulators are already available with different characteristics including the most popular: Netsim^28^ SynTReN^29,^ and GeneNetWeaver.^30, 31^ But in our experience, their simulating engine based on ordinary differential equations (ODEs) resolution is quite slow when solving very large network dynamics and steady states. We thus undertook (i) a different and simpler formalism to routinely simulate and infer large networks, and (ii) to answer very biologist-driven questions.

In this work we use FRANK to simulated GRNs and related gene expressions and use machine learning algorithms to learn back the simulated network structure to benchmark the quality of the reconstruction. However, instead of studying the machine learning algorithms themselves, we rather focused on the impact of the structure of the network, as well as the characteristics of the data needed to perform good reconstruction. In this sense, our work is a very much biologically oriented and proposes math-derived hypothesis to answer the following questions: To what extent prior-knowledge of a given GRNs would be able to improve machine-learning procedures? What amount of prior knowledge is needed to properly infer a GRN of a given size? Which kind of expression data (dynamic, steady state, mixed) are the most valuable to infer a given GRN? Which kind of prior-knowledge (TF-centered or Target-centered) would be best suited to supervise inference of GRNs? What proportion of TF or target gene expression are needed to properly infer GRNs? Are machine learning procedures resilient to bad quality prior knowledge in inferring GRNs from it? Herein, we propose answers to these questions derived from our in silico simulations.

**Fig. 1:**
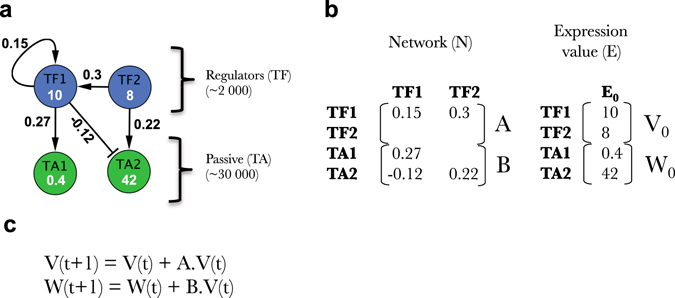
Overall organization of the FRANK algorithm (Fast Randomizing Algorithm for Network Knowledge). **a** Network graph for four genes (two transcription factors [TF]) and two targets (TA). Because of its simple architecture FRANK is able to simulate very large networks (accepting several thousands of TF and TA) and associated gene expression. The model accepts positive, negative and auto-regulations of TFs. **b** The Network graph in panel A is formalized as a network matrix named N made of two sub-matrices: A (TF effect on each others) and B (TF effect on TA). Gene expression at step 0 is then randomized (*E*_0_) made of two sub-vectors: *V*_0_ (being the expression values of TF) and *W*_0_ (being the expression values of TA). **c** Formulas used to iteratively simulate gene expressions across iterative “time points” *t*. Gaussian noise simulating experimental transcriptomic measurements is added

This paper presents the results into two complementary parts. The first one describes FRANK the simulator and the machine learning procedure according to a mathematical/computer science perspective. The second one is biologically oriented and proposes to answer the abovementioned questions. The second part has been built to be independently read by biologists when the first part will require more mathematical skills (except the two first paragraphs describing FRANK general concept related to Fig. 1, see below).

## Results

### Part I: Mathematical and computational simulation

#### Preview of FRANK: a large network simulator

To quickly simulate GRNs of very large size, as well as to control any aspects of the algorithm for further work, we created a simulation algorithm using the C++ language. FRANK formalism is meant to be simple and deterministic to quickly calculate gene expression for very large simulated GRNs (Fig. 1, see FRANK manual for full description provided in Sup. Info. 1).

FRANK is a software that produces (i) GRNs with features considered as crucial in GRN literature (ii) synthetic gene expression values drawn semi-randomly in accordance with the previously built network. Several input parameters (essentially parameterizing probability distributions related to the network features and detailed below) are tuned by the user or provided with default values. The outputs are files containing (i) the simulated network (.csv), (ii) gene expression levels generated by this network (.csv) and heatmaps (.png).

FRANK was designed to quickly generate several hundreds of different large networks having different tunable parameters and their corresponding simulated expression. We have in mind to proceed further with machine learning algorithms, and evaluate the effect of changing GRNs parameters on their learning capacity (second part of the work below).

#### Network and dynamical model

The network is considered here as a directed graph (potentially weighted) and modeled as a large dimensional sparse matrix. This means that each gene is seen as a vertex and interaction between two genes appear as an edge with either a positive (+) or a negative (−) sign depending on the nature of this influence. Depending on the kind of model required, we may then consider two situations: purely directed graphs (edges take values ±1); or weighted graphs (edges are drawn randomly from a Gaussian distribution; for details see Sup File 1 Manual). The network graph (Fig. 1a) is encoded by a network matrix named N, containing two sub-matrices named A and B (Fig. 1b). The sub-matrix A contains TF→TF edges and is squared. B contains TF→TA (Stands for target) edges and is not squared. All the vertices of the graph appear as the row names of the matrix N. Thus N contains null, positive and negative coefficients. A null cell, at line TG (TG can be a TF or a TA) and column TF means that no connection exists from TF to TG. The non-null cells correspond to the edge of the graph mentioned above and are drawn from a Gaussian distribution N(β,1) where parameter β is given. Additional properties of the network are commented below.

Once the network is designed, we can turn to generating the expression levels for the genes through a dynamical process that should be simple for computational reasons but likely to mimic the reality of biological complexity. Let X(t) be the vector of gene expression at time *t* decoupled in two subvectors *X*(t) = (*X*_TF_(t), *X*_TG_(t)) where *X*_TF_(t) denotes the vector of TF expressions and *X*_TG_(t) stand for the expression of TG. We then assume that the margins of X are all log-normally distributed with mean *µ* and standard deviation *σ* ^2^ and perturbed by a measurement error denoted *ɛ* following a centered Gaussian distribution and decomposed in accordance with X hence:

$${X_{{\rm{TF}}}}\left( t \right) = {\rm{exp}}\left( {V\left( t \right)} \right) + {\varepsilon _{{\rm{TF}}}}\left( t \right)$$

$${X_{{\rm{TG}}}}\left( t \right) = {\rm{exp}}\left( {W\left( t \right)} \right) + {\varepsilon _{{\rm{TG}}}}\left( t \right),$$

where V(*t*) and W(*t*) are log of gene expression (see Fig. 1b), with N(*µ*, *σ* ^2^) distribution and follow in addition the evolution equations:

$$\begin{array}{l} V\left( {t + 1} \right) - V\left( t \right) = A \cdot V\left( t \right)\hfill \\ W\left( {t + 1} \right) - W\left( t \right) = B \cdot V\left( t \right)\left[ {{\rm{Model}}} \right]\\ \end{array}$$

Here the square matrix A plays the role of an infinitesimal generator hence contains the information needed to ensure the stability of the system, especially through its eigenvalues.^32^ Designing A and B is consequently at the core of FRANK. The system is fully determined by initial value V(0) and W(0) or equivalently X(0) (we take *ε*(0) = 0) that may be either provided by the user or randomly generated by FRANK. In the sequel the word “iteration” stands for the operation X(*t*) → X(*t* + 1).

#### Main features and calibration

**Fig. 2:**
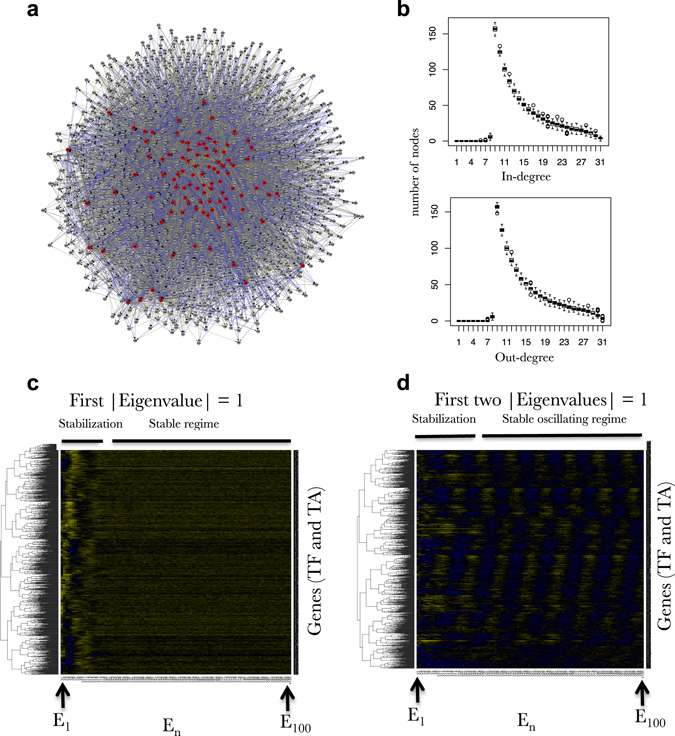
Example of a FRANK outputs (100 TF, 1000 TA). **a** Cytoscape view of a FRANK simulated network having scale free properties. The nodes represent the genes the edges represent the connections in the network (*Blue*: repressive, *Yellow*: inductive). **b** Example of in-degree and out-degree distributions harboring scale-free properties of 100 simulated networks containing 1000 TF and 1000 TA each. **c** Heatmap representations of simulated gene expression from a network (100 TF, 1000 TA) having one Eigen value forced to be equal to 1, **d** Heatmap representations of simulated gene expression from a network (100 TF, 1000 TA) having two Eigen values equal to 1. This heatmap exactly corresponds to the expression of the network depicted in (**a**)

**Fig. 3:**
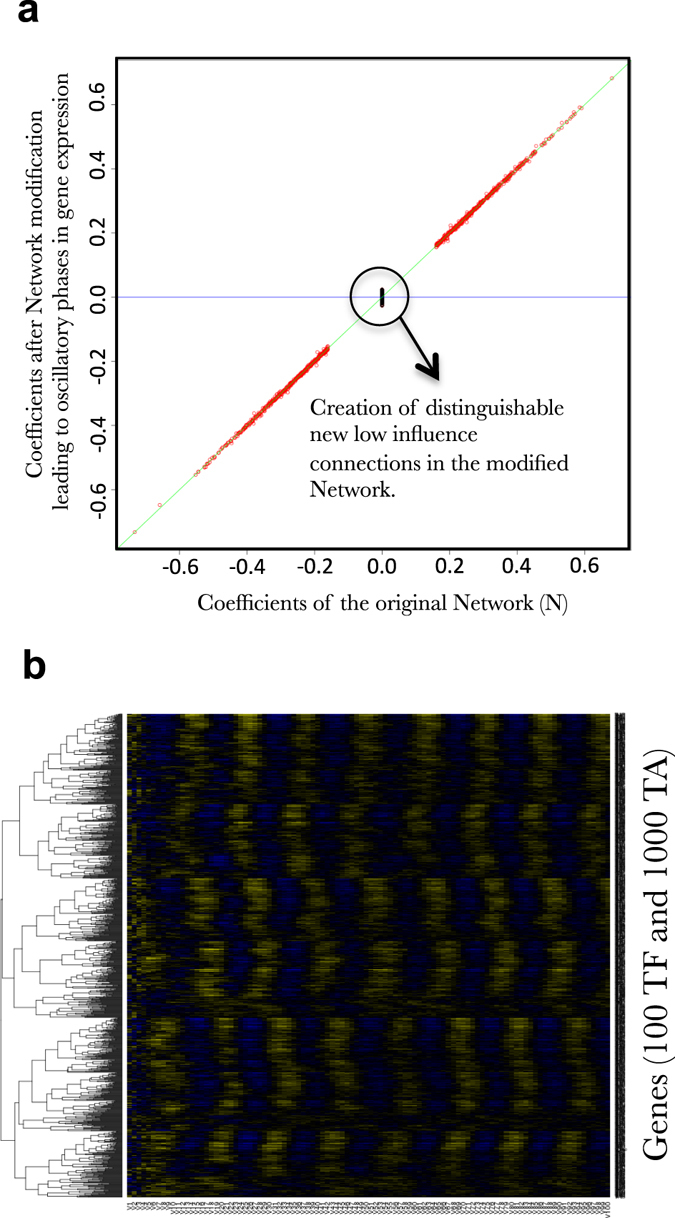
Low TF influences/connections are predicted to be important to drive network expression oscillations. **a** Plot of the coefficient values of the matrix N (the network) before (*x*-axis) and after (*y*-axis) modification (see text) to force 3 Eigen values to be on the unit circle the other one being inside it. **b** Simulated gene expression across 100 iterations (*E*_0_ to *E*_100_) of a FRANK simulated network (100 TF, 1000 TA) displays oscillatory behavior

The network structure can be parameterized for several features. In particular the user can choose a given sparsity, a minimum and a maximum number of TFs controlling a given gene. The network structure is also constrained to harbor scale free properties (see Manual). In silico experiments are then computed in parallel (following the simple formula Fig. 1c) by using a fast exponentiation algorithm based on the dyadic decomposition of the power number (see Manual for full details, Sup. Info. 1). Figure 2 reports some examples of FRANK outputs for a simulated network containing 100 TF and 1000 TA. First, FRANK simulated GRNs display the required network parameters including in and out-scale free properties (Fig. 2b). It is important to note here that, even if the network is built to comply with defined parameters (as mentioned above), its coefficient filling is randomized. Thus, for any raw matrix N built by FRANK, the probability of having a network whose gene expression will be stable across iterations is extremely low. We however assume that network expression stability is a prerequisite to sustain a viable organism. We thus implemented an algorithmic correction of the matrix N to have it generate stable gene expression. This implementation is related to the complex eigenvalues of A (*n*th eigenvalue arranged in a decreasing order of moduli is termed *λ*_n_). More specifically the location of the eigenvalues with respect to the unit circle in the complex plane is crucial: all the eigenvalues should be inside the disk to ensure convergence, at least one must be on the unit circle to ensure stability and more than one located in the unit circle if one seeks for oscillations and periodicity of the system. But this stability condition has to be managed within a sparse matrix framework. We found no specific work that addresses the issue of complex eigenvalue location (within the unit disk) for large sparse matrices arising in gene regulatory network. Our solution consists of a small perturbation of the (m-sparse) matrix constructed earlier with IN and OUT-scale free properties. First, all coefficients are standardized so that *ρ*(A) = 1 with *ρ*(A) the spectral radius of matrix A. This does not change sparsity or any initial properties of the matrix. Then we compute its eigenvalues. Since A is real, these eigenvalues are real or conjugate. We select an integer say *p* < 10 that accounts for the complexity required in the network. We pick the 2p conjugate eigenvalues closest to the unit disk (they are necessarily inside the circle) and move them vertically-up for the one with positive imaginary part and down for the other-until they reach the unit disk. This operation leads to a new matrix, say, A′ with A′ = A + *E*_p_, where *E*_p_ depends only on the eigenvectors related to the 2p conjugate eigenvalues considered above and on the small purely imaginary perturbation that projects the eigenvalues onto the unit circle. The resulting A′ is still a real matrix bit loses its sparsity in a strict mathematical sense. Switching from A to A′ leads to new coefficients with very low but non-null values (see Fig. 3a). We observe a clear gap between these new network connections of low influence and the coefficients of the original network. These new connections are likely to be necessary to observe stable oscillatory behavior in gene expression. This observation is further discussed below for its potential biological consequences (see Part II).

#### Learning versus inference

Along the past 20 years, statistical science provided several reliable methods for studying gene regulatory network. The standard statistical tools used to address this problem are based on the reconstruction of the network using gene expression data. We mention here that ODE systems, standard in GRN modeling, stem from distinct areas of mathematics and with different goals.

Network reconstruction may be split into two different approaches. Most of the techniques infer the network: roughly speaking they try to discover all the edges simultaneously from gene expression.^26, 33^ Our approach here differs substantially. Indeed, we do not apply inference algorithm but rather intend to literally learn the network (even if the word “learning” is now abusively used for inference methods such as LASSO).

More specifically we assume here, that we are given the exact structure of a piece of the network (prior knowledge). We then train a learning algorithm on the known part and finally try to predict the unknown part of the network. The dichotomy between inference and learning that we underline here is important for us not just because it involves different techniques, but also because it opposes interpretability and predictability. We do not seek an easy-to-understand (sparse or causal) model but the best possible network prediction. The price to pay relies on using black-box methods and also on concerns in calibrating/tuning the parameters. FRANK appears as a useful tool to carry out pure learning procedures. We describe now shortly four learning algorithm known for their reliability (LASSO, decision trees, deep neural networks (NN), support vector machine (SVM)) and explain the reasons why we lastly selected the SVM.

#### Four benchmarks methods for gene network inference and learning: reasons for selecting SVM

The LASSO is a penalized mean square program with l1 penalty (see the historical reference).^34^

$${\rm{mi}}{{\rm{n}}_\beta }\mathop {\sum}\limits_{i = 1}^n {{{\left( {{y_i} - {\beta ^{\rm{T}}}{X_i}} \right)}^2} + \gamma {{\left| \beta \right|}_1}} ,$$

where *γ* is the regularization parameter. The LASSO consequently estimates a linear regression model with an additional constraint on the 11 norm of the slope vector. It has well-known thresholding properties: the selected slope parameter usually features several zero coefficients. In other words when the tuning parameter *γ* is chosen to be large enough, *β* may have a large number of null coordinates. The LASSO is very well suited to the sparse data encountered in gene regulation network and may be computed with low complexity algorithm but has some drawbacks. Indeed, an underlying model is assumed and this model is linear, the LASSO estimate, although providing nice interpretation properties, has poor prediction power and is suited for network inference, whereas, as explained earlier, we are fundamentally going through a learning approach (refer to^35^ for deeper information about the LASSO).

Classification trees and random forests are other robust methods. Briefly speaking, a classification is a tree where each node provides a combination of input variables and each leaf is associated with a class of the output variable (here *y*). At each step, for each node, an input variable is selected according to its ability to split the sample in the best possible way. This ability is measured by quantitative criteria such as Gini impurity, entropy or variance deflation. The inherent complexity of the resulting trees is balanced by pruning the tree. Pruning is usually carried out by examining the cross-validation error. Several extensions to decision trees were proposed in order to improve their performances: bagging, boosting, and random forests are the most popular.^36^ Conversely to the LASSO, classification trees and their extensions by ensemble methods were designed for learning and for use in a prediction approach. However in our framework,^37^ of the original data was definitely a stumbling stone and we could not carry out decision tree or random forests correctly on data with sparsity levels as observed in biological networks. Indeed, these approaches happen to run for several days on our servers without leading to any interesting results.

NN are more and more popular since they proved their efficiency in image analysis. They consist in building a sequence of nonlinear processing (each element of this sequence is called a layer) to detect informative features in the data. The layer N processes the features computed at stage N−1 and is expected to refine them. The final result may be viewed as a hierarchy of representation for the data and may be carried out either for clustering or classification purposes. In our framework we tested several architectures of deep (with fewer neurons) or non-deep (with many neurons) NN. We also carried two classical strategies for pretraining: Stacked Denoising AutoEncoder and restricted Boltzman machines. Our experience shows that deep networks do not outperform single layers networks whenever a sufficient number of neurons is involved. Besides it is not clear to us that NN are the best tool to cope with the sparse information structure of GRN.

Another issue arises. Indeed when supervised learning is carried out, these NN need large amounts of data. Since here we intend to address the question of reconstructing the network from a minimal prior knowledge, NN were outperformed by the fourth and last method presented below.

At last we introduce SVM slightly more deeply than the three previous methods. SVM are another popular method for classification by machine learning.^38, 39^ Consider a two class problem and suppose that we are given a training dataset {(*X*_1_, *y*_1_),(*X*_2_, *y*_2_),…,(*X*_*n*_, *y*_*n*_)} where *X*_*i*_ is a vector in R^p^ and *y*_*i*_ is either −1 or +1 depending on the class *X*_*i*_ belongs to.

We can state first the mathematical setting in the simplest framework. Imagine that the training dataset may be perfectly separated by a hyperplane (Sup. Fig. 1[SvmPlot.pdf]) the sample depending on two variables *x*_1_ and *x*_2_. For red triangle points the class is (*y* = −1) and for blue circles the class is (*y* = +1). Here the SVM computes the equation of the straight line that splits the two groups in an optimal way. Optimal here means that the corridor (dotted lines) around the straight line is the largest possible. The three filled points are called “support points” because the computation of the optimal hyperplane depends only on the points located on the edge of their groups.

We can write now the SVM program with mathematical symbols:

$${\rm{mi}}{{\rm{n}}_{b0,b1}}\left\| {{b_1}} \right\|,{\rm{subject}}\,{\rm{to}}\quad {y_i}\left( {{b_1}{\;}^T{X_i} + {b_0}} \right) \ge 1,$$

where $y = {b_1}{\;}^TX + {b_0}$ is the hyperplane equation. It can be shown then that 1/||*b*_1_|| is proportional to the “corridor width”. The constraints appearing on the right hand side of the equation above just reads “points such that *y* = +1 are on one side of the hyperplane and points such that *y* = −1 are on the other side.

The description of the classification problem above is very specific at least for three reasons. First, we assumed that two groups are strictly separated which is not true in general. Second, we take it for granted that both groups may be linearly separated. It is not hard to think of situation, where the frontier between the two groups may be a quadratic or exponential function or equations of other kinds. Third, if we turn back to the gene network problem, we should consider three possible valued for *y*, namely 0 (no edge), +1 (activation) and −1 (inhibition).

When the groups are not strictly separated—which means that some points of, say group (*y* = −1) are mixed with points of group (*y* = +1)—the program above may be adapted by relaxing the constraint. It suffices to replace ${y_i} \left( {b_1}{\;}^T X_{i} + {b_0}\right)  \ge 1$ by ${y_i} \left( {b_1}{\;}^T{X_i} + {b_0} \right) \ge 1 - {c_i}$ where *c*_*i*_ stands for the gap between the current point and its group’s margin.^40^

The non-linear generalization of SVM is surprisingly not that intricate, and it essentially relies on the use of specific kernels and on Reproducing Kernel Hilbert space (RKHS) theory. Let *K*( . , . ) be a positive kernel defined on the design space (here R^p^) and denote H the RKHS associated to K.

The general and abstract SVM program is given below.

$${\rm{mi}}{{\rm{n}}_{b0,f}}{\left\{ {{{\left\| f \right\|}_H}{\;}^2 + \theta \mathop {\sum}\limits_{i = 1}^n {{c_i}} } \right\}_{{\rm{ject}}}}{y_i}\left( {f\left( {{X_i}} \right) + {b_0}} \right) \ge 1 - {c_i},{c_i}  >0\left[ {{\rm{SVM}}} \right]$$

Above all the *c*_*i*_ and *θ* are positive, the latter being a tuning parameter. Given a new design point ***x***, the decision rule stems from *y*(*x*) = *sgn*(*f*(*x*)+*b*_0_) where *f* is a solution of the program above and may always be written under the form:

$$f\left( x \right) = \mathop {\sum}\limits_{i \in S} {{a_i}{y_i}K\left( {x,{x_i}} \right)} ,$$

where *S* denotes the set of active points (i.e., the points that match the constraints in [SVM]). The SVM program comes down to estimating the coefficients *a*_*i*_ above subject to the dual program of [SVM].

Although the choice of the kernel is rarely crucial we tested Gaussian (RBF) kernel vs. several other kernel types: polynomial of order 1 and 2, Bessel, etc. We kept the latter in all our work because it involves a single-bandwidth or variance-parameter. This bandwidth is selected by a cross validation approach.^41^ The other tuning parameter is the Lagrange multiplier interpreted as the cost for constraint violation. It was set to 1 in accordance with strategies often carried out with SVM.

Finally the connection with multi-classes SVM, that is when *y* takes more than two values, is achieved by specific algorithm that reduces this issue to multiple binary problems.^42^

#### Learning on FRANK generated data

After choosing a proper model and selecting the best method (SVM) the last step in our methodology consists in evaluating the learning process. To that aim we consider here essentially two scores that have a biological meaning and are insightful and classical for practitioners: the percentage of true positive (non-null edge detected as non-null including the direction of the regulation [positive or negative]), and the percentage of false positive (null edges detected as positive or negative) (see Part II).

#### A trick for data selection

In our early investigations we faced a problem stemming from the sparse structure of the data. The learning algorithm mentioned above was not designed to cope with sparse data. The output values may possibly take values −1, 0 and 1 but we observed that whatever the method at work the predictions are essentially null. As a consequence we often faced the issue of constant (null) predicted values that is a very low rate of true positive. Improving the ability of the methods to detect vertices was a challenge. We introduced a trick that is inspired from boosting that artificially increases the proportion of positive (vertices) in the learning sample.

-Simulate a *n*-sample of data and collect the output values (*y*_1_, *y*_2_, …, *y*_*n*_),

-Keep those *y*_*i*_′s that are −1 or + 1, denote *n*_≠_ the cardinal of this set of output values $V^{*} = \left( {y_1^*,y_2^*, \ldots y_{{\rm{n}} \ne }^*} \right),$

-Select at random n_≠_ amongst the *n*−*n*_≠_ remaining null *y* data denoted

$${V^0} = \left( {y_1^0,y_2^0, \ldots y_{{\rm{n}} \ne }^0} \right),$$

-Perform the learning algorithm on *V* ^0^∪*V* ^*^.

The four steps above tend to remove the sparsity in the learning data at the expense of a serious decrease of the sample size. Clearly the proportion of 50% zeros/50% non-zeros may be tuned to other values.

This somewhat unusual though pragmatic change turns out to enhance predictions and meet the goals of increasing the true positive rate (Sup Fig. 2).

### Part II: Biological insights using FRANK and SVM

#### Benchmarking the role and the characteristics of prior knowledge and transcriptomic data to improve supervised machine learning of GRNs

In Part I, we defined FRANK (Fig. 1) as a rapid and effective large network simulator. FRANK provides (i) network modules having the characteristics that are observed in eukaryotic systems (Figs. 1 and 2) and (ii) simulated gene expression in a large number of conditions (Fig. 2).

The challenge then was to learn the network module using SVM applied on: (i) simulated transcriptomic data generated by the network and (ii) a set of given connections of the network (prior-knowledge, called alpha in the following Figures). Indeed, as defined in the introduction, many techniques are now available to actually experimentally probe GRNs (eY1H, ChIP-Seq, TARGET, DAP-seq…) that may be used to improve our GRN learning capacities. The particularity of our approach was also, not to prove that prior-knowledge was important (it as already been demonstrated),^24^ but rather study the characteristics of this prior-knowledge as well the characteristics of the transcriptomic data.

**Fig. 4:**
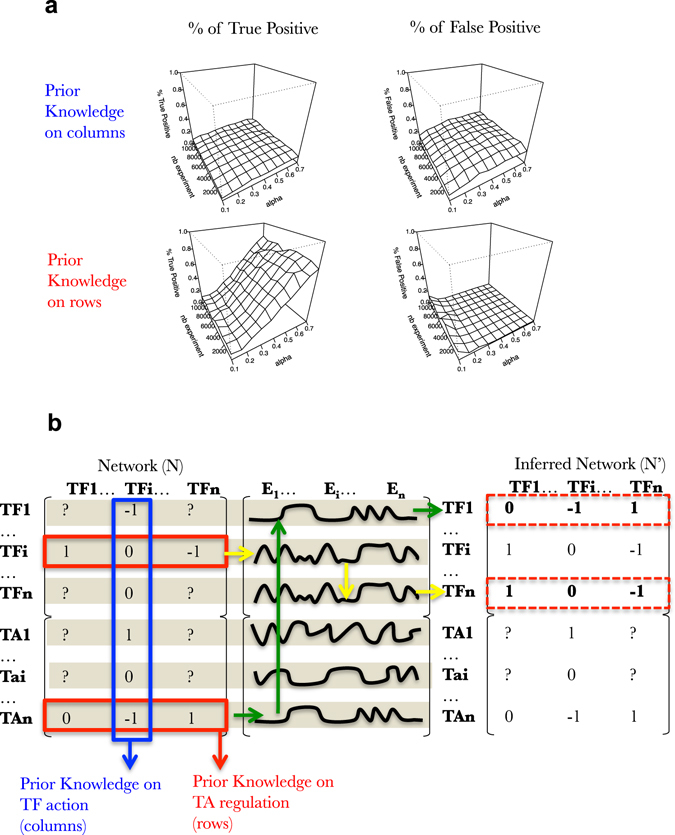
TA-oriented prior knowledge is superior to TF-oriented prior in order to supervise SVM machine learning on simulated data. **a** Surfaces exploring the SVM accuracy (% of True positive on *left* and % False positive in *right*) to predict the connections in a FRANK generated network (100 TF, 1000 TA) using an increasing number of simulated gene expressions (*nb experiment*; *y*-axis) and an increasing fraction of the network as prior knowledge (*alpha*; *x*-axis). Percentage of true positive and false positive are evaluated based on predictions of the presence of an edge and its positive or negative influence. Simulated gene expression data kind: A^1^. **b** Schematic of the selection of data for supervision of machine learning procedure (here SVM). In *blue*, columns are selected to serve as prior knowledge to supervise SVM. This may correspond to experimental techniques using one TF that explore its exhaustive activity (binding via ChIP-Seq, or GR-fusion studies for instance). In *red*, rows are selected to serve as prior knowledge to supervise SVM. This may correspond to experimental techniques using one particular target gene/promoter that explores its exhaustive attractiveness (i.e., Y1H studies). *Yellow* and *Green arrows* are used to explain the process of machine learning (see text)

**Fig. 5:**
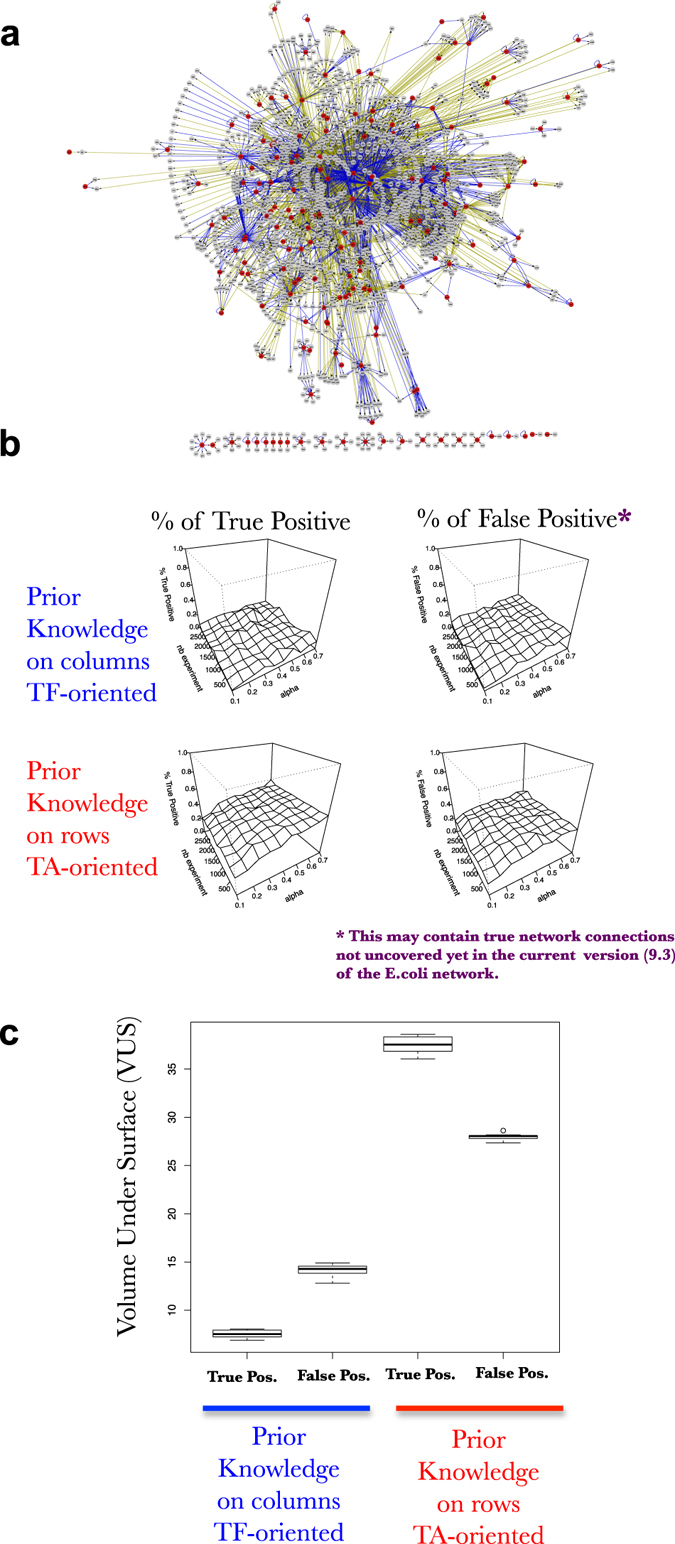
TA-oriented prior knowledge is superior to TF-oriented prior in order to supervise SVM machine learning on real data. **a** Cytoscape display of the current *E. coli* network (version 9.3 retrieved from http://regulondb.ccg.unam.mx/) **b** Surfaces exploring the SVM accuracy (% of true positive on *left* and % false positive in *right*) to predict the connections in *E. coli* network (171 TF, 1493 TA) using an increasing number of real gene expressions (*nb experiment*; *y*-axis) and an increasing fraction of the network as prior knowledge (*alpha*; *x*-axis). Percentage of true positive and false positive are evaluated based on predictions of the presence of an edge and its positive or negative influence. **c** Bootstrap results for ten learning processes as the one described in **b**. For each bootstrap cycle 90% of the network has been resampled and genes expression randomized. The panel represents the boxplot of VUS for the ten learning procedures

**Fig. 6:**
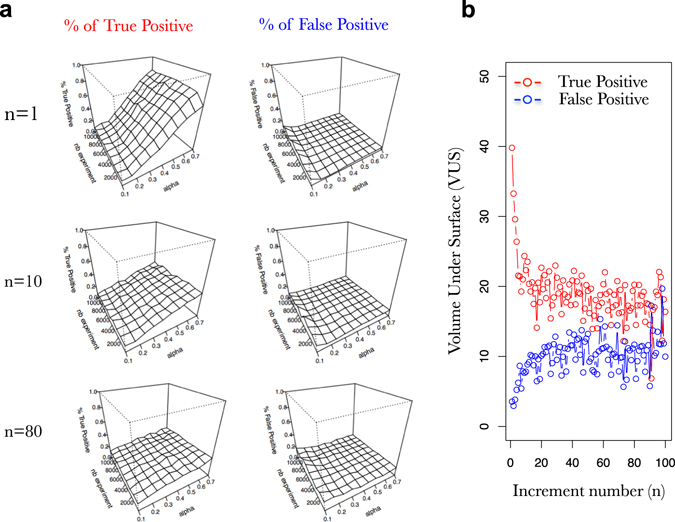
Small increment in gene expression are more powerful to properly learn the network with SVM and prior knowledge than steady states. **a** Surfaces exploring the SVM accuracy (% of true positive on *left* and % false positive in *right*) to predict the connections in a FRANK generated network (100 TF, 1000 TA) using an increasing number of simulated gene expressions (*nb experiment*; *y*-axis) and an increasing fraction of the network as prior knowledge (*alpha* [rows are used as prior knowledge]; *x*-axis). Percentage of true positive and false positive are evaluated based on predictions of the presence of an edge and its positive or negative influence. **b** Progression of the volume under the surface (as in **a**) computed for a progression in (A + I)^*n*^ for the % of true positive (*red*) and the % of False positive (*blue*) predicted connections in the network. Simulated gene expression data kind: multistart (A^1^, …, A^100^); prior knowledge data kind *rows*

**Fig. 7:**
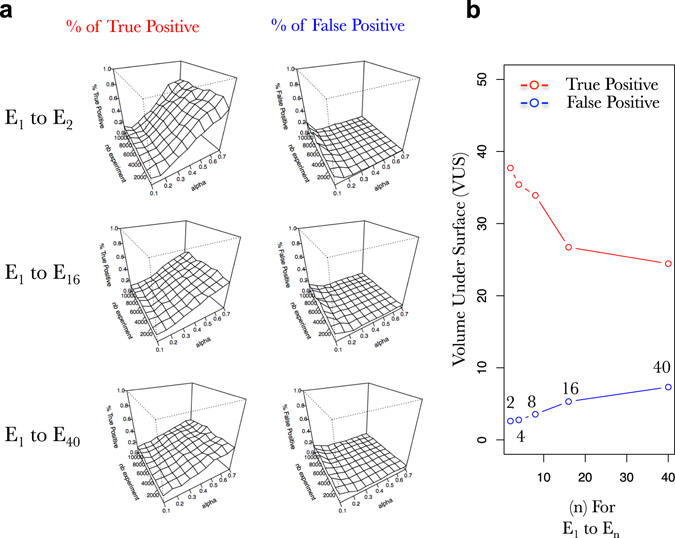
Early short dynamics in gene expression are more useful to properly learn the network than longer ones. **a** Surfaces exploring the SVM accuracy (% of true positive on *left* and % false positive in *right*) to predict the connections in a FRANK generated network (100 TF, 1000 TA) using an increasing number of simulated gene expressions (*nb experiment*; *y*-axis) and an increasing fraction of the network as prior knowledge (*alpha* [rows are used as prior knowledge]; *x*-axis). Percentage of true positive and false positive are evaluated based on predictions of the presence of an edge and its positive or negative influence. **b** Progression of the volume under the surface (as in **a**) computed for a progression in (A + I)^*n*^ for the % of true positive (*red*) and the % of False positive (*blue*) predicted connections in the network. Simulated gene expression data kind: dynamic (E_1_ to E_*n*_); prior knowledge data kind *rows*

**Fig. 8:**
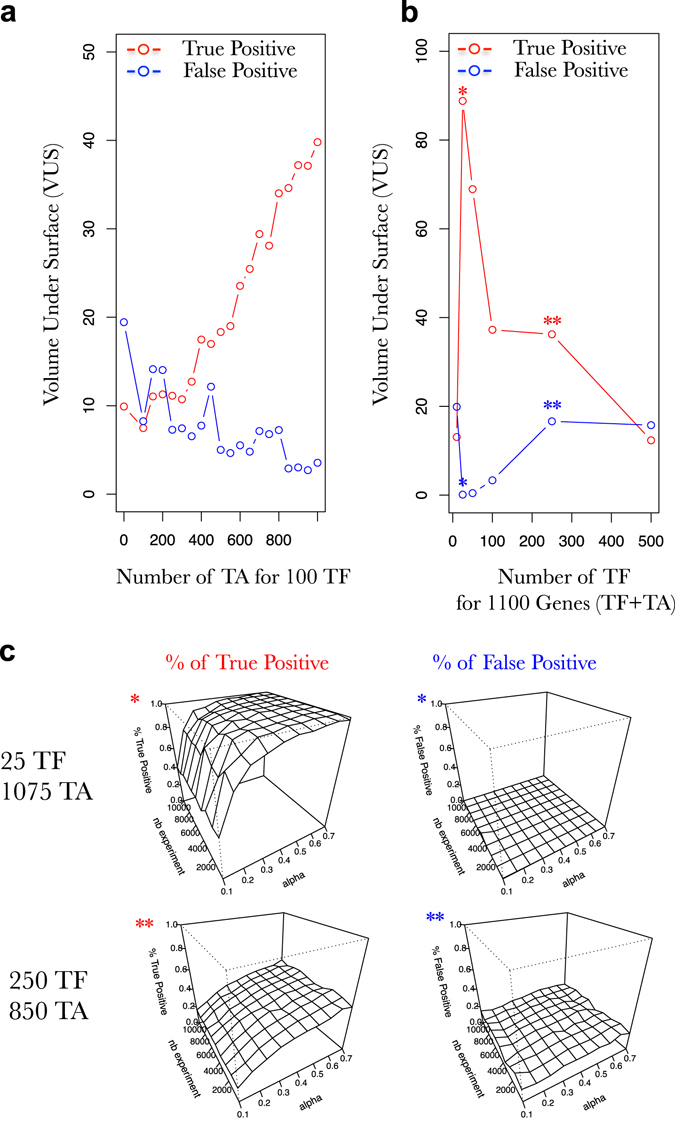
Target genes (TA) are important for the accurate reconstruction of the network. **a** Progression of the volume under the surface computed for a progression in the number of TA for FRANK generated networks having a constant number of 100 TF. **b** Progression of the volume under the surface computed for a progression in the number of TA for FRANK generated networks having a constant number of genes (TA + TF = 1100). *Red*, % of True positive and *blue*, the % of false positive predicted connections in the network. Transcriptomic data kind: A^1^; prior knowledge data kind *rows*. **c** Example of surfaces exploring the SVM accuracy (% of true positive on *left* and % false positive in *right* panels) to predict the connections in a FRANK generated network (25 TF, 1075 TA for the top 2 surfaces and 250 TF, 850 TA for the bottom two surfaces) using an increasing number of simulated gene expressions (*nb experiment;* y axis) and an increasing fraction of the network as prior knowledge (*alpha* [rows are used as prior knowledge]; *x*-axis). Percentage of true positive and false positive are evaluated based on predictions of the presence of an edge and its positive or negative influence. The surfaces presented in **c** correspond to two particular points in the **b** noted by *asterisks*

**Fig. 9:**
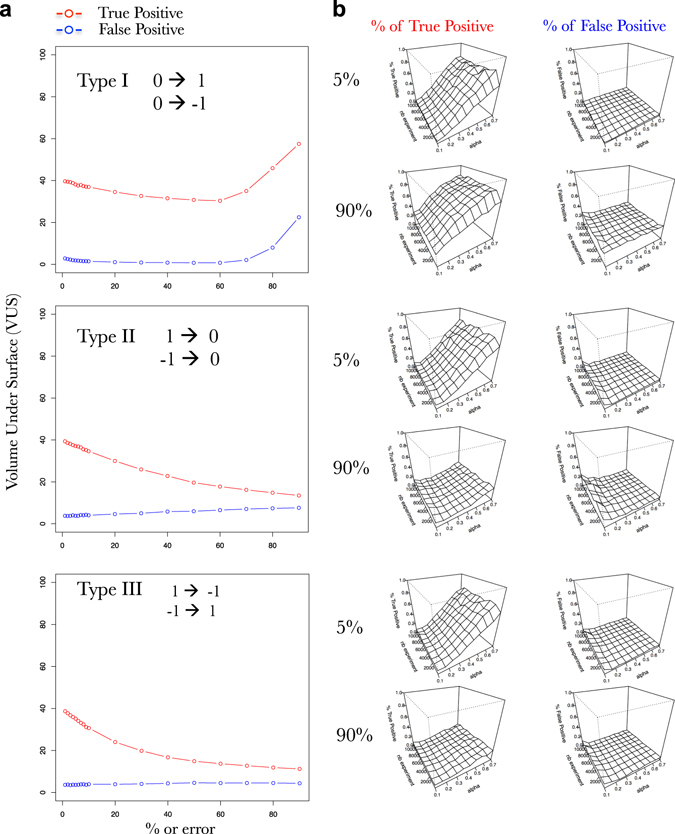
SVM learning is resilient to prior knowledge containing mistakes of type I. **a** Three error types have been defined and proposed to SVM: type I (simulates false positive data); type II (simulates false negative data); and type III (wrong-sign influences). Progression of the volume under the surface computed for a progression of % of Type I–III errors fed as prior knowledge. **b** Surfaces exploring the SVM accuracy (% of true positive on *left* and % false positive in *right*) to predict the connections in a FRANK generated network (100 TF, 1000 TA) using an increasing number of simulated gene expressions (*nb experiment*; *y*-axis) and an increasing fraction of the network as prior knowledge containing errors of three different types (*alpha* [rows are used as prior knowledge]; *x*-axis). True positive (*red*) and the % of false positive (*blue*) predicted connections in the network. Percentage of true positive and false positive are evaluated based on predictions of the presence of an edge and its positive or negative influence

**Fig. 10:**
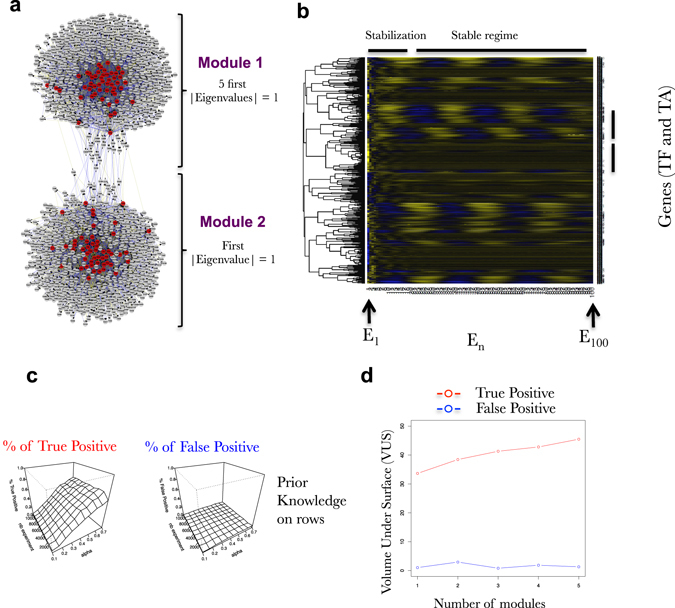
Network modularity does not compromise SVM learning capacities. **a** Modular networks have been built by combining two network modules harboring different stability behavior. The first one displays stable non-oscillating behavior (First |Eigen Value| = 1), the second one stable and oscillating genes expression (five first |Eigen Value| = 1). The two modules are connected by their hubs. **b** Heatmap representations of simulated gene expression from a network (100 TF, 1000 TA) composed of two modules of 50 TF and 500 TA each. Each module has different stability behavior plain stable or oscillating. This heatmap exactly corresponds to the expression of the network depicted in (**a**). **c** Surfaces exploring the SVM accuracy (% of true positive on *left* and % false positive in *right*) to predict the connections in a FRANK generated network (100 TF, 1000 TA) using an increasing number of simulated gene expressions (*nb experiment*; *y*-axis) and an increasing fraction of the network as prior knowledge (*alpha*; *x*-axis). Percentage of true positive and false positive are evaluated based on predictions of the presence of an edge and its positive or negative influence. Simulated gene expression data kind: A^1^

We develop this point in following sections. For each section we evaluate the accuracy of SVM learning according to its capacity to uncover connections and their directions (positive or negative corresponding to positive or negative sign of the coefficient in the network N). In this work, we only evaluate if a particular TF controls a particular gene, which is so far what is needed in biology. Mathematically, this corresponds to reconstructing the support of the network N plus the direction of the edges and not the coefficient value per se. This evaluation is made base on two metrics: the % true positive, and the % of false positive (see for example Fig. 4a). The first one corresponds to the percentage of the correct non-null predicted edges. The second corresponds to the percentage of non-null predicted edges that are actually equal to 0. These two values are respectively expressed throughout the manuscript as a surface reporting their relationship between the number of experimental data points (understand simulated transcriptomic experiment) and the prior knowledge needed expressed in % of the network N (Fig. 4a). When discrete changes are evaluated such as in Figs 5–10, an additional metric is computed which corresponds to the volume under the surface (VUS) which is conceptually close to the popular area under the curve (basically, a two-dimensional extension).

#### Oscillatory phenomena at a whole network scale are predicted to require a decrease in network sparsity and widespread influence of TFs genome wide

Before starting with the machine learning procedure, we first emphasize a discovery made when developing FRANK itself. Indeed, Fig. 3a presents the relationship between the coefficients of the network matrix (*N*) before and after Eigen value correction that produces gene expression stability in an oscillatory mode (Fig. 3b). It is stunning to observe that the overall correction does not dramatically affect the coefficient values of *N*. Indeed, we observed a very low dispersion around the diagonal. However, it may be interesting to note that the correction leading to oscillation is creating new connections of very low influences that seem needed to maintain the stable oscillatory behavior. Furthermore, these new connections of low influence can still be distinguished from the pre-existing connections (before Eigenvalue correction). Mathematically, as evoked above, this means that strictly speaking, the high degree of sparsity of the network *N* seems to be incompatible with the generation of the oscillatory gene expression at the whole genome level. Biologically, this would mean that TFs involved in the oscillatory modules, controlling large networks (up to 80% of the genome display oscillations in plants for instance),^9^ should display a high degree of connectivity but with potentially very low influence on many genes. This prediction may find some echoes in the recent experimental ChIP-seq investigations of the central regulatory TF controlling circadian oscillations in plants, named CCA1. Indeed, CCA1 ChIP-seq results demonstrated that this TF is bound to more than 1000 genomic regions representing approximately 4–5% of the genes in the genome.^9^ Our model may explain to some extent such an important widespread influence of a key oscillator. This may experimentally reveal mathematical constraints on overall network structure to reach stable oscillations.

#### TA oriented prior knowledge is predicted to be superior to supervised SVM machine learning procedures to learn GRNs

To begin with, we asked whether (i) prior knowledge is likely to improve GRNs learning and (ii) what kind of prior knowledge is the most appropriate to supervise SVM. To do this, we simulated network containing 100 TFs and 1000 TAs. For each network gene expression was simulated following a “multistart” logic (fully explained and studied below for its effect on learning). Surfaces Fig. 4a are computed to report true positive and false positive as a function of the number of experiments (ranging from 1000 to 10,000) needed and a fraction of the prior-knowledge on the network (varying from 0.1 to 0.7).

What we observe here is that, as expected, the accuracy of network learning increases with the number of data points provided (Fig. 4a, Sup Fig. 2A, 2B). More interestingly, prior knowledge has a strong effect on SVM learning capacity not only by improving the percentage of true positive (left hand side) but also by strongly decreasing the level of false positive. This observation opens very important perspectives when it comes to solving real GRNs using experimental data.

Furthermore, we observe that the prior knowledge quality also strongly influence the learning capacity of SVM (compare the two top surfaces to the two bottom one Fig. 4a). Indeed, two kinds of prior knowledge can exist (see Introduction). The first one is TF oriented, where a TF is used to probe its overall genomic activity or binding (ChIP-Seq, TARGET or DAP-seq). Herein, we will refer to this as TF oriented or prior knowledge on columns (because it uncovers a column in the Network N, Fig. 4b). The second kind is TA oriented, where one can find the TFs that bind or regulate a particular promoter (eY1H screens for instance). We will refer here to this as TA oriented or prior knowledge on rows (because it uncovers a row in the Network N, Fig. 4b). Strikingly, we have found that the TA oriented prior knowledge is largely superior to the TF oriented prior knowledge when it comes to supervised SVM learning (Fig. 4a). This was rather counterintuitive at the first look, but can be easily explained as follows. In Fig. 4, we schematized the learning process that we applied. We can see that the SVM machine learning process is seeking for commonalities in the gene regulation. If two genes display a strong correlation such as TF_i_ and TF_n_, or TA_n_ and TF1 in Fig. 4b, the machine learning process will look for the prior knowledge available for these particular genes and apply this knowledge to infer the missing information (N′) (follow yellow or green arrows in Fig. 4b). If this prior knowledge is TF or column oriented a lot of missing information will appear for a particular gene (Fig. 4b). Conversely, if the prior knowledge is TA or row oriented, one can imagine that the prior knowledge is quite complete to fill in the inferred network N′.

To our knowledge this observation was original and we wanted to evaluate if this predicted influence of the prior-knowledge structure was also evident when SVM are provided with real data (real network and real transcriptome). To this end, we decided to use one of the currently “best electronically encoded regulatory network of any free-living organism”, *E. coli* K-12. We retrieved the network from the database http://regulondb.ccg.unam.mx/, (Regulon v9.3) and several thousands of transcriptomic data points (http://m3d.mssm.edu/norm/ ) as described in Fu et al. work.^43^ The network was recoded to be compatible with our pipeline of analysis. We built a *N* matrix with −1.0 + 1 influences. The reformatted network contains 171 TF, 1493 TA, connected by 4195 regulatory edges (Fig. 5a). Following the same pipeline of analysis as before on simulated data (Fig. 4 for example), we ran SVM on real network and real transcriptomic data (Fig. 5b). Despite the fact that here, we used the entire transcriptomic dataset and that the provided network may still holds imperfections, we observed that TA-oriented prior knowledge is indeed more useful than TF-oriented prior to train SVM (Fig. 5b). We applied bootstrap techniques in order to evaluate the fact that % of True positive are systematically higher than the % of False positive in the case of TA-oriented prior knowledge, as reported by VUS (Fig. 5c). This demonstrates that prior structure influences SVM learning even on real datasets.

In conclusion, from simulation (Fig. 4) and from real network and transcriptome data (Fig. 5), we believe that for the same amount of data points the eY1H or similar TA oriented approaches are likely more powerful to train machine learning than TF oriented techniques. Of course, we believe that having more data points will always improve learning when this will be applied to real datasets and that mixed (TA and TF-oriented) prior knowledge will finally be used to decipher GRNs. But if one needs to invest in having more data to supervise GRN machine learning, our results show that it might be more helpful to carry out TA oriented techniques.

#### The first steps in gene expression preceding stable regime contain the information needed to learn GRNs

In this part, we also evaluated the kind of gene expression that contains the more information to best learn GRNs using prior knowledge. Indeed, FRANK uses an iterative process to generate gene expression data (See part I, Fig. 1). Here, this iterative process starts with the randomization of the gene expression E_0_ at step 0. Then the model is applied once to reach E_1_ (expression of the genome at step 1). Here, we can distinguish between two ways of simulating genome expression.

The first one is named “multistart” (close to Monte Carlo in spirit), where the above process is repeated *n* times by sorting out a new E_0_ each time.

The second one is named “dynamic”, where E_1_ is used to reach E_2_ and so on and so forth up to E_*n*_ (this progression is the one being displayed in Figs. 2c, d, 3b). These two concepts will be used right below.

To understand what are the characteristics of the transcriptome that may contain the most information for GRN learning, we decided to build gene expression datasets containing values of multistart process reaching the step E_*n*_ (Fig. 6). For instance for *n* = 10 the experiments provided to the SVM learning are a compilation of E_10_ genome expression for many different E_0_. What has been observed here is that the smallest increments in gene expression are the most useful to learn the network. Indeed, Fig. 6a shows that the learning capacity of a supervised SVM is clearly more efficient if one uses E_1_ instead of E_10_. This is clearly exemplified by the VUS progression in response to increasing n values presented Fig. 6b. Indeed, we recorded a very marked decrease in the SVM capacity to learn the GRN from *n* = 1 to *n* = 10 that is manifest at the same time because of a decrease in the true positive as well as an increase in the false positive (Fig. 6b). This means that the most useful information in the transcriptomic dataset for GRNs learning lies in the fast response following gene expression perturbation. When the gene expression reaches its steady state it would be very difficult to learn the underlying GRNs. This is a pretty intuitive result but we believe that our approach provides a clear measurement and simulation of such phenomenon.

Starting from this above observation, we wanted to simulate gene expression that may resemble more to the dynamics that are provided by real transcriptomic datasets. In other words, it is quite unusual to perturb cellular networks and harvest a particular time point many times (such as in Fig. 6). Actually biologists usually perform kinetics.^44–47^ This means that perturbation is applied once and then samples are harvested across time. This is what we wanted to explore next. Thus, in Fig. 6 we performed dynamics of different sizes and measure what is the most useful to train supervised SVM. Again we observe that dynamics can be used to solve the network with a pretty good accuracy (Fig. 6) in particular with shorter dynamics (Fig. 6b). Interestingly, when a dynamic including the first 16 iteration steps (corresponding to the stabilization regime in Fig. 2c, d) is used, the supervised SVM still performs with a good accuracy (Figs. 6a and b). This result (i) opens very interesting perspectives concerning the applicability of this supervised learning on real datasets (ii) provides a good entry point to the relationship between the mathematical iteration and the real life time scale (see Discussion) and has been discussed and studied by others.^44–47^

#### TF/TA ratio matters in supervised GRN learning

The network named N is virtually built of two sub networks named A and B (Fig. 1). A contains all the TF to TF relationships, whereas B contains all the TF to TA relationships. Thus, one of our preconceptions of the system at the beginning of this study was that A is likely to process the information when B is only receiving information from A (Fig. 1). Thus, according to this, in a first instance, one could imagine that solving or learning A would be enough to understand the whole network N. We actually found that, when the supervised learning is applied this idea is actually wrong.

To evaluate the role of TA genes in the machine learning process, we decided to generate several FRANK networks having variable TF/TA ratios by (i) keeping the number of TF constant (100) and increasing the number of TA (Fig. 7a), (ii) keeping the number of genes constant (1100) and varying the TF and TA number (Fig. 7b). In both cases, we observed that by increasing the TF/TA ratio the GRN learning efficiency decreases (Figs. 7a, b). Very strikingly, we even reached a very peculiar point where the learning is nearly perfect (Fig. 7c) with a network having 25 TF and 1075 TA. It is perfect in a sense where with a relative low number of experiments (~2000) and a relatively low level of prior knowledge (0.3), SVM reach nearly 80% of true positive and produce no false positive connections. We are perfectly aware that this situation is far from being what is found in real networks. However, we believe that this peculiar point is very informative concerning the potential of supervised SVM learning when applied to sub-networks. Furthermore, this demonstrates that TA information is very important to reconstruct the whole network. The explanation likely lies into what we have developed above concerning TA-oriented prior-knowledge (Fig. 4b).

#### Supervised machine learning algorithms are predicted to be robust to prior-knowledge errors

In the previous parts of this work, we established that prior-knowledge, in particular TA-oriented one, are key to supervised SVM and may radically help to reconstruct GRNs in a near future. Nevertheless, in the previous simulations, all the prior knowledge that we used to supervise the learning processes contained 100% of true connections. However, in wet lab experimental conditions, it is quite known that the results of ChIP-seq, Y1H, or DAP-seq are likely to contain false positive or false negative results. We thus, wanted to test how resilient (robust) might be the supervised learning if errors where introduced in the prior-knowledge. To do so, we simulated three types of errors (Fig. 8). Type I errors create a certain number of false connections in the prior knowledge, having positive or negative influences. Type II errors remove a certain percentage of the actual connections in the prior knowledge. Type III errors change the directions of a certain percentage of the connections in the prior-knowledge. We thus tested the capacity of SVM to learn the actual network even though the prior knowledge was changed and noisy. Very interestingly, we observed that supervised SVM are resilient to any kind of error up to 10%. Furthermore, we observed that supervised SVM are particularly resilient to type I errors as compared to type II and type III (Fig. 8a). This can be first explained by the sparsity of the network. Indeed, biological networks seem dense (Fig. 2a) but their actual connections represent a very little portion of all the possible connections between the nodes. Hence, the network (N) is mathematically sparse (have a lot of 0). Thus, when we provide 20% or error for instance, it still represents a small proportion of the real connections. Furthermore, because the errors that we make are sampled randomly the probability for having the same error reproduced for two different genes is quite low. Thus, by the same principle explained above (Fig. 4b), it is very likely that the SVM is detecting the artificially introduced errors.

#### Network modularity does not impact learning capacities of supervised SVMs

In the preceding parts of this work, we focused on the learning of network modules having homogenous properties (Fig. 2a). However, biological networks are expected to be modular.^48, 49^ A module is by definition, a discrete entity whose function is separable from other modules but likely receiving signals from these latters. We thus first wanted to implement FRANK simulation towards the production of modular networks. We also wanted to evaluate the effect of modular networks on the machine learning capacities of supervised SVMs. To build a modular network we combined 2 FRANK stable networks (one being plain stable and the other one being oscillating) (Fig. 9a, b, see FRANK manual for details Sup. File 1). The two modules are linked together by their hubs (most connected TFs in each modules) following the general observation of hierarchical networks.^49^ We were able to indeed connect two network modules displaying two different intrinsic behaviors in their gene expression though connected via some TFs (Fig. 10b). We then evaluated the learning capacities of SVM on this modular network. We found that network modularity does not impact learning capacities of SVM (Fig. 10c). We further applied this logic for an increasing number of modules (number of TF and TA being constant). We again found that network modularity does not impact learning capacities of SVM for a modularity being higher than two (Fig. 10d). Indeed, we observed that learning on row-oriented prior-knowledge performed as efficiently on non-modular (Fig. 4a) as compared to modular networks (Fig. 10c). In both cases (modular and non-modular) column-oriented prior-knowledge dramatically decreased the learning capacities of SVM. This demonstrates that modularity (i) does not change the conclusions drawn concerning the structure of the prior-knowledge and its influence on learning; (ii) may not be a limitation to supervised learning procedures applied to real datasets as results in Fig. 5 may confirm.

## Discussion

In his famous essay published almost 40 years ago^50^ Francois Jacob describes evolution as a tinkering rather than an engineering process. We embrace this vision. Thus GRNs that we are now observing in nature are intrinsically the outcome of an iterative try-and-selection/Darwinian process. However, reverse engineering procedures can also be of great interest to understand the possibilities offered by nature to design biological objects. This is in line with the famous Richard Feynman sentence: “What I cannot create, I do not understand”. We believe that the creation of FRANK shed some light on probable design principles of GRNs. Indeed, herein we employed reverse engineering to delineate the potential properties of big (containing thousands of genes and ten of thousands connections) GRNs. Doing so we uncovered an interesting feature concerning large network sparsity and stability. Indeed, we have observed that to obtain a stable network displaying oscillatory behaviors, we need to force at least two Eigenvalues to be on the unit circle. This observation is not a novelty in the mathematical field of dynamical systems, however it is to our knowledge the first time that this concept is related to large gene network modeling. This observation also point further towards an important potential inverse relationship between sparsity and oscillatory stability in gene expression (Fig. 3). This is a prediction of our models and it needs to be experimentally observed. This would mean that TFs are likely to have many subtle influences on a large portion of the genome, and that influence is important to maintain gene expression oscillatory stability. Some experimental observations in nature are in accordance with this fact (see above).^9^

Another important issue that rises from our studies is the relationship between the iterative process that we modeled, and the real time scale in cell biology. Indeed, when we observe individual gene behavior during the stabilization phase of gene expression (Figs. 2c, d) it resembles very much what we can observe during transcriptomic analysis following treatments: genes are regulated and sometimes display an overshoot which then reaches a stable state. In nature this overshoot in gene expression can be observed within ~20 min with a stability phase happening within a couple of hours.^44^ This can vary according to the biological model, the perturbation of the network, and the GRN studied. This means that E_20_ (Figs. 2c, d) is likely to correspond to hours of treatment. Consequently we evaluate the simulated step in our iteration process to be equivalent to ~5–6 min of cell response (120 min/20 steps). But this constitute a rough calibration that will deserve further work.

When it comes to study of our capacity to learn the GRN by using supervised SVM, we wanted to draw some general conclusions that we believe can help to design future machine learning procedures on real datasets. We found that (i) short dynamics are more powerful to teach machine learning algorithms than longer ones (Figs. 6, 7), (ii) prior knowledge greatly helps SVM to define real underlying GRNs even if it contains errors (Figs. 1–10), (iii) prior knowledge is far more efficient when it represents Target-oriented results (Figs. 4 and 5, such as Y1H for instance), (iv) studying the whole network connectivity in particular by studying a lot of passive genes (TA) is important for the learning process (Fig. 8). From these observations, even if the cell system is over-simplified in our model, we really believe that by using this approach to teach the machines the actual true connections in the network, we will be able to accurately reconstruct GRNs in a near future.

We have also found that prior knowledge not only greatly improves detection of true positive connections, but also strongly decreases the percentage of false positive. Take Fig. 4a for instance. For 35% of prior knowledge on rows the SVM algorithm is able to reconstruct 40% of the rest of the network. This may appear to be quite low. However, it is very important to note that, after 35% of prior knowledge the algorithm produces nearly no false positive (Fig. 4a right surface). Meaning that out of 40% of network reconstruction mainly all the connections are true ones. This would mean that one could use this newly discovered connections as new prior knowledge to further train the SVM in a next cycle of the learning process. This approach, termed boosting in computer science,^51^ will certainly be an important aspect of future GRN learning algorithms.

Concerning limitations, one needs to be aware that FRANK is an over-simplified version of transcriptional GRNs. In particular the coefficient in the network are fixed across iterations, which otherwise could be related to the influence of post-transcriptional and post-translational modifications. Furthermore, it is important to note that even if the FRANK formalism uses a sort of discrete linear system (Fig. 1), the TF to TA relationships; *X*_TG_ = *f*(*X*_TF_) in part I, are not linear but polynomial, which does not prevent the relationship to be of sigmoidal form as often observed in nature.

Finally, we would like to bring the attention of the reader on one aspect, which makes our approach reasonable. On one hand, the FRANK formalism has helped to discover that TA-oriented prior-knowledge is likely to be more informative to train SVM than TF-oriented prior-knowledge (Fig. 4a). On the other hand, since (i) this conclusion holds true for real network learning (Fig. 5) and that (ii) the explanation of the phenomenon stems into the inherent structure of FRANK system (Fig. 4b), we conclude that the FRANK formalism bring us a bit further towards the reality of GRNs in cells, despite its obvious limitations.

## Methods

The modeling and machine learning procedures (FRANK) are fully described in the manual of the algorithm provided online (Sup File 1). The model equations are fully described in the Result section Part I. The learning procedures have been implemented on a DELL server using SVM package (*Kernlab*) on R (https://www.r-project.org/).

### Data availability

FRANK software can be used online via a web page (https://m2sb.org/ ? page = FRANK) or scripts will be provided upon request to any of the authors.

## Electronic supplementary material

Supplementary File 1

Supplementary Figure 1

Supplementary Figure 2
