# Supplementary material for: Reverse engineering highlights potential principles of large gene regulatory network design and learning
Source: NPJ Syst Biol Appl. 2017 Jun 22;3:17. doi: 10.1038/s41540-017-0019-y (PMC5481436; doi:10.1038/s41540-017-0019-y)
Supplement: Supplementary file 1 [file 41540_2017_19_MOESM1_ESM.pdf]

FRANK

1 Creation of the TF matrix

The TF matrix creation algorithm as used in FRANK follow 4 steps:

1. We fill a $TF \times TF$ dimensional matrix A with zeros.

2. We select a few positions (i, j) (in order to respect sparsity) where we put random values in accordance with the following scheme.

   - The randoms values are simply realisations of $\mathcal{N}(0, 1)$, moved away from 0 by a given parameter β.
   - In order to simulate a *scale-free* network, we use the following algorithm to choose the non-zero positions:
     (a) We compute the probability distribution of sparsity by row/column, following an exponential distribution of parameter *alpha* truncated from *sparsityMin* to *sparsityMax*.
     (b) By multiplying the probabilities by the number of row/column (TF) we obtain the average number of filled row/column of a given sparsity.
     (c) We compute the total number of non-zero elements.
     (d) We allocate a row and a col vectors where at each coordinate we associate a sparsity value following the previously computed distribution.
     (e) We randomize the order of elements for each of this two vectors.
     (f) While this total number of elements is non-zero we apply the following pattern[1]:
        i. We select the indexes of the row vector where it is non-zero, we call this index set *indexByRow*
        ii. We do the same for the column vector and obtain an *indexByCol* index set.

[1] We are aware that this algorithm will not return a strictly distributed scale-free matrix, there is a possibility for the same set of coordinate to be selected multiple times. In practice this problem arises for small matrices where the sparsity is of the same scale as the number of elements. For large matrices, very sparse, like transcription networks, the final distribution is indiscernable from a scale-free distribution.

iii. we choose uniformly at random an index *irow* in *indexByRow* and *icol* in *indexByCol* thus obtaining a $(irow, icol)$ position where we put a random value in the matrix.

iv. We then decrement by 1 the value at *irow* for the vector row and the value at *icol* for the vector col (These indices will not be selected again if reaching the zero value).

v. We decrement by one the total number of non-zero elements.

3. Once the matrix filled, we add 1 to its diagonal elements (in order to satisfy the $A + I$ constraint)

4. We then adress the eigenvalues problem: we want to have a matrice with a given number, $nUnitEigenVal$, of eigenvalues in the unit circle.

   (a) We have to ensure that our matrix can support the transformation[2] we will apply: we need that the $nUnitEigenVal^{th}$ and $(nUnitEigenVal+1)^{th}$ eigenvalues (by decreasing order of absolute value) are *not* conjugate eigenvalues. If our matrix does not meet this condition, we simply reroll a new one until satified.

   (b) Once we have a good matrix, we compute its eigenvalues.

   (c) We divide all cells of the matrix by the norm of the largest eigenvalue (in complex norm) in order to have all the eigenvalues inside the unit sphere.

   (d) We store the eigenvectors of this matrix.

   (e) For each eigenvalue, from the first up to the $nUnitEigenVal^{th}$ we compute the minimal translation in the complex axis needed to have this eigenvalue on the unit circle.

   (f) We then translate the eigenvalues to effectively put them on the unit circle.

   (g) Finally we *compute back* our matrix using our new eigenvalues and the initial, stored, eigenvectors. The resulting matrix will not be sparse anymore, but the *old zero* coordinate are small and the fact that we moved away from zero the *real non-null* values makes us able to still differentiate between the two. We need the values to be different from zero from a stability point of view.

2 Creation of the TG matrix

The creation of the TG matrix (a.k.a the B matrix) is simpler, because we do not need to check for the out-interactions to be distributed as a power law (there is no out-interactions for this genes). The designing algorithm is as follows:

[2]By supporting the transformation, we mean that the resulting matrix should be real and not complex after the transformation. If the conditions are not met, after transformation we will have a complex eigenvalue without conjugate, resulting in a complex valued matrix.

- We compute the probability distribution of sparsity by row (the in-interactions) as the distribution for the matrix A (exponential).
- For each row of the matrix, we draw at random, following the computed distribution.
- For this row, knowing the number of non-zero elements, we draw their column indexes at random as a uniform distribution.
- The value set at each position is computed exactly as for the A matrix: a $\mathcal{N}(0,1)$, moved away from 0 by a given parameter β.
- Finally we divide all the non-zeros values of the matrix by the highest eigenvalue of A computed previously in order to keep the same scale between the two matrices.

3 Parsing the experiments

We use an homebrew code for expressing experiments, the specifications are as follow:

- An experiment is a set of *histories*.
- Each history is assigned a *starting vector*.
  - You can provide a file name containing a set of starting vectors.
  - If no starting vectors are provided, or if there is not enough starting vectors, FRANK will randomly generate the right quantity of vectors.
- Each history is enclosed in curly braces { }, sometimes followed by a *multiplicativity token*.
- A multiplicativity token is composed of the * symbol followed by a number of repetition of the same history.
- Each repetition is considered as a different history (but they share the same pattern of observations), and thus have different starting vectors.
- An history is composed of *observations* separated with space or tabular symbols.
- An observation is a number representing a point in the discret modelisation of the time and can be of three kind:
  1. A single number. for example: 100
  2. A range of numbers represented by two integers separated by a minus - symbol. The range goes from the first argument to the second with a step of 1 between each observations. For example: $1 - 100$

3. A range of numbers represented by three numbers separated by minus - symbols. The range goes from the first argument to the third with a step given by the second number. For example: $1 - 2 - 100$

As an example, the following experiment: {1 5 100}*5 {1 5-5-100} is composed of 6 histories (six different starting vectors). The first to the fifth history follow the same pattern which is: observations 1, 5 and 100, and the sixth history follows a different pattern which is: observations 1, 5, 10, 15, 20, ..., 95, 100.

4 Computation

1. In order to save computation time, the computation is done in parallel, also we use a fast exponentiation algorithm based on the diadic decomposition of the power number.
   - We store in the variable K the maximum observation value we need to compute.
   - For each t from 1 up to $k = \min\{p \in \mathbb{N} | 2^{p+1} - 1 \geq K - 1\}$ we compute and store the A^{2^t} matrix.[3]
   - For a given observation with value t, we compute the diadic decomposition of $t - 1$ and multiply only the necessary A^{2^k} matrices with the starting vector. We start with the vector and we apply the matrices in order to the resulting vector, thus effectively using only a minimal number of matrix $\times$ vectors operations at the price of the storage of $p + 1$ matrices in memory.
2. The dyadic decomposition is also applied to the modified versions of the matrices if needed.
3. We apply A and B (or the modified versions) to the resulting vector, obtaining the desired result.
4. Finally we apply the *log-normal* transformation

$$lognormal(x) = e^{x\sqrt{var_{data}} + mean_{data} + \mathcal{N}(0, \sqrt{var_{noise}})} \quad (1)$$

to each coordinates of the vector, the result being a log-normal distributed vector.

5 Modularity

Each generated GNR computed by FRANK is *per se* a single module. We were able to bring modularity in GNR by simulating different iterations of FRANK and assembling them in a single network matrix. We choose to connect the

[3]We check only for $K - 1$ instead of K because we apply separatedly the final A or B matrix as needed.

different sub-networks (modules) by the most connected points (hubs) of the matrices.

- The first step was to generate two sub matrices, one for each module.
- We then create a new matrix with the two modules in his diagonal.
- We find the n (parameter) greatest hubs for each module.
- We choose p (parameters) points of connection. Each connection is uniformly chosen (without replacement) between the two set of greatest hubs (we connect hub to hub).

The resulting matrix will effectively behave like a two module GNR.
Once two modules have been connected, we can iterate this pattern using the modular network and a new module to combine them in a new three-module network. This can be generalized to build network with an arbitrary module size.
