# Supplementary material for: Reverse engineering highlights potential principles of large gene regulatory network design and learning
Source: NPJ Syst Biol Appl. 2017 Jun 22;3:17. doi: 10.1038/s41540-017-0019-y (PMC5481436; doi:10.1038/s41540-017-0019-y)
Supplement: Supplementary file 2 — Supplementary Figure 1 [file 41540_2017_19_MOESM2_ESM.pdf]

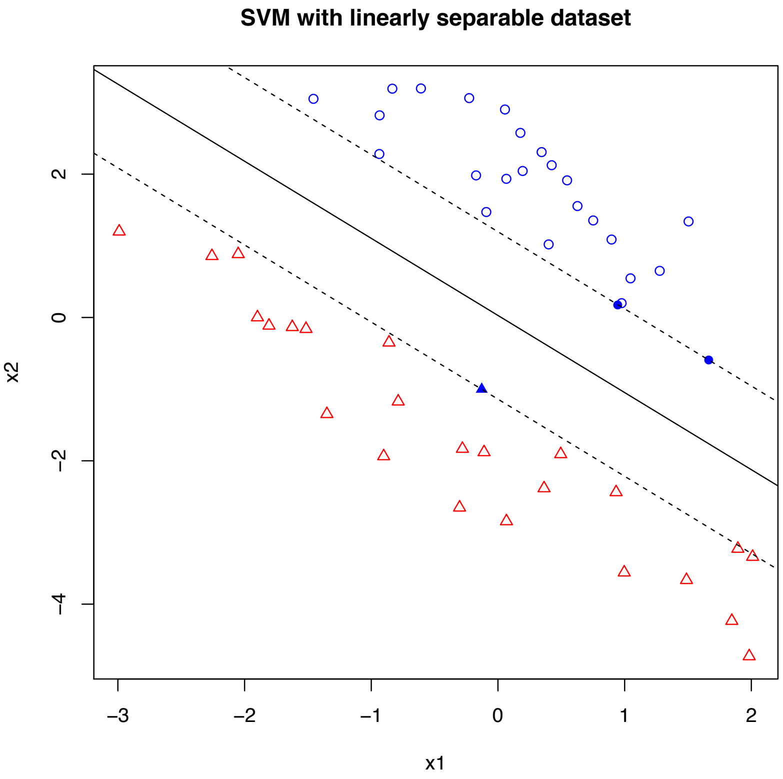

Supplementary Figure 1. Illustration of the SVM logic. On this example the two sets of points (triangle versus circles) may be exactly seperated. The three support vectors (1 triangle, 2 circles) are coloured in blue. They are landmarks that determine the dotted line corridor. The separator hyperplane (here the black straight line) splits the corridor in two equally spaced margins.
