# Supplementary material for: Reverse engineering highlights potential principles of large gene regulatory network design and learning
Source: NPJ Syst Biol Appl. 2017 Jun 22;3:17. doi: 10.1038/s41540-017-0019-y (PMC5481436; doi:10.1038/s41540-017-0019-y)
Supplement: Supplementary file 3 — Supplementary Figure 2 [file 41540_2017_19_MOESM3_ESM.pdf]

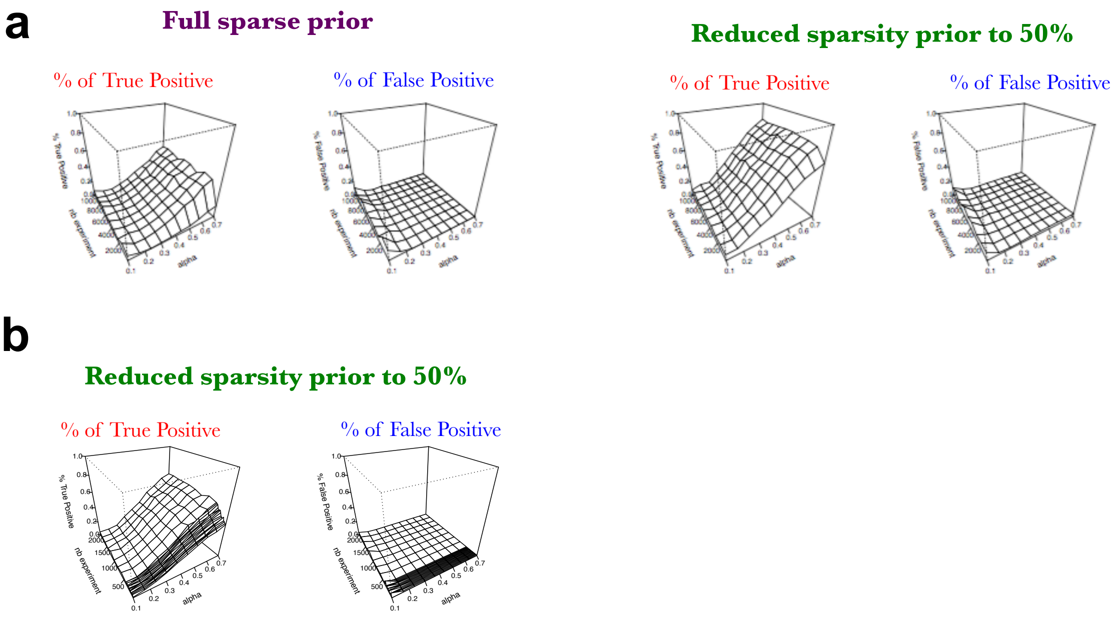

Supplementary Figure 2. Data selection on prior knowledge reducing sparsity improves machine learning predictive capacity of SVM.

Surfaces exploring the SVM accuracy (% of True positive on left panels and % False Positive in right panels) to predict the connections in a FRANK generated network (100 TF, 1000 TA) using an increasing number of simulated gene expressions (*nb experiment;* y axis) and an increasing fraction of the network as prior knowledge (*alpha* [rows are used as prior knowledge]*;* x axis). Percentage of True positive and False positive are evaluated based on predictions of the presence of an edge and its positive or negative influence. (A) number of experiments vary from 1000 to 10000. (B) number of experiments vary from 100 to 2000.
